# Correction: Determining the factors of m-wallets adoption. A twofold SEM-ANN approach

**DOI:** 10.1371/journal.pone.0314906

**Published:** 2024-12-02

**Authors:** Imdadullah Hidayat-ur-Rehman, Saeed Alzahrani, Mohd Ziaur Rehman, Fahim Akhter

There are errors in the Author Contributions. The correct contributions are:

**Conceptualization:** Imdadullah Hidayat-ur-Rehman, Saeed Alzahrani, Mohd Ziaur Rehman, Fahim Akhter.

**Data curation:** Imdadullah Hidayat-ur-Rehman.

**Formal analysis:** Imdadullah Hidayat-ur-Rehman.

**Methodology:** Imdadullah Hidayat-ur-Rehman.

**Writing–original draft:** Imdadullah Hidayat-ur-Rehman, Saeed Alzahrani, Mohd Ziaur Rehman, Fahim Akhter

**Writing–review & editing:** Mohd Ziaur Rehman, Fahim Akhter.
