# Supplementary material for: Determining the factors of m-wallets adoption. A twofold SEM-ANN approach
Source: PLoS One. 2022 Jan 28;17(1):e0262954. doi: 10.1371/journal.pone.0262954 (PMC8797175; doi:10.1371/journal.pone.0262954)
Supplement: S1 Text — (DOCX) [file pone.0262954.s001.docx]

**Measurement Items**

**Relative Advantage** adopted from (Kaur et al., 2020)

RA1: M-wallets have more advantages than internet or cash payment systems.

RA2: M-wallets are more convenient than internet or cash payment systems.

RA3: M-wallets are more efficient than internet or cash payment systems.

RA4: M-wallets are more effective than internet or cash payment systems.

**Compatibility** adopted from (Kaur et al., 2020)

COMP1: Using an m-wallet is compatible with all aspects of my lifestyle.

COMP2: Using an m-wallet is completely compatible with my current situation.

COMP3: I think that using an m-wallet fits well with the way I like to buy.

COMP4: Using an m-wallet fits into my lifestyle.

**Ease of Use** adopted from (Liébana-Cabanillas et al., 2018)

EOU1: It is easy to become skilful at using m-wallets

EOU2: Interactions with m-wallets are clear and understandable

EOU3: It is easy to follow all the steps to use m-wallets

EOU4: It is easy to interact m-wallets

**Observability** adopted from (Kaur et al., 2020)

OB1: I have seen others using m-wallets.

OB2: I have often seen others using m-wallets

**Trialability** adopted from (Kaur et al., 2020)

TR1: I know more about new products before other people do.

TR2: I am usually among the first to try new products.

**Convenience** adopted from (Chen et al., 2019)

CONV1: I believe that using m-wallet will be convenient.

CONV2: I think that it is easy to use m-wallet to accomplish my payment tasks.

CONV3: Using M-wallet saves my time.

CONV4: Compared to traditional payment methods, I believe that m-wallet methods are more convenient.

**Personal Innovativeness** adopted from (Schmidthuber et al., 2020)

PI1: If I heard about a new information technology, I would look for ways to experiment with it.

PI2: Among my friends/colleagues, I am usually the first to try out new information technologies.

PI3: I like to experiment with new information technologies.

**Perceived Security** adopted from (Matemba & Li, 2018)

PS1: The application offers a safe environment to send sensitive information through.

PS2: I believe the application has security measures to send sensitive information through.

PS3: I believe the transaction details in the application is protected.

PS4: I would feel totally safe to provide my credit/debit card to purchase products through the application.

**Perceived Trust** adopted from (Matemba & Li, 2018)

PT1: I trust the application.

PT2: I think the application always provides accurate financial services.

PT3: I think the application interests my mind.

PT4: I think the application always provides secure financial services.

**Intention-to-use MW** adopted from (Kaur et al., 2020)

IU1: I expect my use of m-wallets to increase in the future.

IU2: I intend to use m-wallets in the future.

IU3: If I have an opportunity, then I will use an m-wallet.

IU4: I will always try to use an m-wallet.

IU5: I plan to use m-wallets frequently.
