# Supplementary material for: Determining the factors of m-wallets adoption. A twofold SEM-ANN approach
Source: PLoS One. 2022 Jan 28;17(1):e0262954. doi: 10.1371/journal.pone.0262954 (PMC8797175; doi:10.1371/journal.pone.0262954)
Supplement: S2 Text — (DOCX) [file pone.0262954.s002.docx]

**Survey Questionnaire**

Dear Respondent

We are a research team from King Saud University, Riyadh and conducting a research on the topic of “Mobile Wallets (M-Wallets) Adoption”. The aim of this survey is to provide insights on adoption of M-Wallets. To achieve this objective, it is essential that all respondents provide an honest assessment of their experience about M-Wallets and fairly answer all the questions. I assure, your answers will remain confidential and will use only for academic purpose.

***Do you use any Mobile Wallet Application?***

- Yes
- No

**Section-I: Demographic Profile**

Please tick the appropriate box.

1. **Gender**

- Male
- Female

1. **Age Group**

- 16 to 25 years
- 26 to 35 years
- 36 to 45 years
- 46 to 55 years
- Above 55 years

1. **Qualification**

- High/Higher School
- Undergraduate
- Graduate
- PhD.

1. **Employment**

- Full-Time
- Part-Time
- Self Employed
- Student
- Retired

1. **Which Mobile Wallet application used?**

- STC Pay
- Mada Pay
- Bayan Pay
- Halalah
- Samsung Pay
- Apple Pay
- PayPal
- WeChat pay
- Other, please specify _______________________

1. **Usage Experience?**

- Less than 6 months
- Between 6 months to 12 months
- Between 1 year to 2 years
- Above 2 years ____________

1. **How frequent do you use m-wallet?**

- Once a day
- Once a week
- Once a month
- Other, please specify

____________

**Section-II: Respondents’ Opinion about M-Wallets**

Please indicate the level of your agreements with the following statements by selecting appropriate number in front of each statement.

| **Strongly Disagree (SD)** | **Disagree (D)** | **Neutral (N)** | **Agree (A)** | **Strongly Agree (SA)** |
| --- | --- | --- | --- | --- |
| **1** | **2** | **3** | **4** | **5** |

| **S. No.** | **Items** | **Level of Agreement** | | | | |
| --- | --- | --- | --- | --- | --- | --- |
|  |  | **SD** | **D** | **N** | **A** | **SA** |
| RA1 | M-wallets have more advantages than internet or cash payment systems. | **1** | **2** | **3** | **4** | **5** |
| RA2 | M-wallets are more convenient than internet or cash payment systems. | **1** | **2** | **3** | **4** | **5** |
| RA3 | M-wallets are more efficient than internet or cash payment systems. | **1** | **2** | **3** | **4** | **5** |
| RA4 | M-wallets are more effective than internet or cash payment systems. | **1** | **2** | **3** | **4** | **5** |
| COMP1 | Using an m-wallet is compatible with all aspects of my lifestyle. | **1** | **2** | **3** | **4** | **5** |
| COMP2 | Using an m-wallet is completely compatible with my current situation. | **1** | **2** | **3** | **4** | **5** |
| COMP3 | I think that using an m-wallet fits well with the way I like to buy. | **1** | **2** | **3** | **4** | **5** |
| COMP4 | Using an m-wallet fits into my lifestyle. | **1** | **2** | **3** | **4** | **5** |
| EOU1 | It is easy to become skilful at using m-wallets | **1** | **2** | **3** | **4** | **5** |
| EOU2 | Interactions with m-wallets are clear and understandable | **1** | **2** | **3** | **4** | **5** |
| EOU3 | It is easy to follow all the steps to use m-wallets | **1** | **2** | **3** | **4** | **5** |
| EOU4 | It is easy to interact m-wallets | **1** | **2** | **3** | **4** | **5** |
| OB1 | I have seen others using m-wallets. | **1** | **2** | **3** | **4** | **5** |
| OB2 | I have often seen others using m-wallets | **1** | **2** | **3** | **4** | **5** |
| TR1 | I know more about new products before other people do. | **1** | **2** | **3** | **4** | **5** |
| TR2 | I am usually among the first to try new products. | **1** | **2** | **3** | **4** | **5** |
| CONV1 | I believe that using m-wallet will be convenient. | **1** | **2** | **3** | **4** | **5** |
| CONV2 | I think that it is easy to use m-wallet to accomplish my payment tasks. | **1** | **2** | **3** | **4** | **5** |
| CONV3 | Using M-wallet saves my time. | **1** | **2** | **3** | **4** | **5** |
| CONV4 | Compared to traditional payment methods, I believe that m-wallet methods are more convenient. | **1** | **2** | **3** | **4** | **5** |
| PI1 | If I heard about a new information technology, I would look for ways to experiment with it. | **1** | **2** | **3** | **4** | **5** |
| PI2 | Among my friends/colleagues, I am usually the first to try out new information technologies. | **1** | **2** | **3** | **4** | **5** |
| PI3 | I like to experiment with new information technologies. | **1** | **2** | **3** | **4** | **5** |
| PS1 | The application offers a safe environment to send sensitive information through. | **1** | **2** | **3** | **4** | **5** |
| PS2 | I believe the application has security measures to send sensitive information through. | **1** | **2** | **3** | **4** | **5** |
| PS3 | I believe the transaction details in the application is protected. | **1** | **2** | **3** | **4** | **5** |
| PS4 | I would feel totally safe to provide my credit/debit card to purchase products through the application. | **1** | **2** | **3** | **4** | **5** |
| PT1 | I trust the application. | **1** | **2** | **3** | **4** | **5** |
| PT2 | I think the application always provides accurate financial services. | **1** | **2** | **3** | **4** | **5** |
| PT3 | I think the application interests my mind. | **1** | **2** | **3** | **4** | **5** |
| PT4 | I think the application always provides secure financial services. | **1** | **2** | **3** | **4** | **5** |
| IU1 | I expect my use of m-wallets to increase in the future. | **1** | **2** | **3** | **4** | **5** |
| IU2 | I intend to use m-wallets in the future. | **1** | **2** | **3** | **4** | **5** |
| IU3 | If I have an opportunity, then I will use an m-wallet. | **1** | **2** | **3** | **4** | **5** |
| IU4 | I will always try to use an m-wallet. | **1** | **2** | **3** | **4** | **5** |
| IU5 | I plan to use m-wallets frequently. | **1** | **2** | **3** | **4** | **5** |

Thank you for your precious time and cooperation.
