# Supplementary material for: Determining the factors of m-wallets adoption. A twofold SEM-ANN approach
Source: PLoS One. 2022 Jan 28;17(1):e0262954. doi: 10.1371/journal.pone.0262954 (PMC8797175; doi:10.1371/journal.pone.0262954)
Supplement: S3 Text — (DOCX) [file pone.0262954.s003.docx]

**استبيــــان**

عزيزي المشارك:

نحن فريق بحثي من جامعة الملك سعود بالرياض ونجري بحثًا حول موضوع "اعتماد محافظ الهاتف المحمول (M-Wallets)". الهدف من هذا الاستبيان هو تقديم رؤى حول اعتماد محافظ الهاتف المحمول (M-Wallets). لتحقيق هذا الهدف ، من الضروري أن يقدم جميع المشاركين تقييمًا صادقًا لتجربتهم حول استخدام محافظ الهاتف المحمول (M-Wallets) والإجابة بشكل عادل على جميع الأسئلة. و نؤكد أن إجاباتك ستبقى سرية وستستخدم للأغراض الأكاديمية فقط.

هل تستخدم أيّاً من تطبيقات محافظ الهاتف المحمول؟

- نعم
- لا

**القسم الأول : ديموغرافية الملفّ الشخصي**

يُرجي اختيار الخيار المنــاسب :

1. الجنس

- ذكر
- أنثى

1. الفئـة العمرية

- من 16 إلى 25 ســنة
- من 26 إلى 35 سنة
- من 36 إلى 45 سنة
- من 46 إلى 55 سنة
- فوق 55 سنــــــة

1. المؤهــل

- الثـانوية العامة
- طالبـ/ـة في المرحلة الجامعية
- طالبـ/ـة دراسات عليا
- دكتوراة

1. الوظيفة

- دوام كلي
- دوام جزئي
- اعمل لحسابي الخاص
- طالب
- متقاعد

1. أيّاً من محافظ الهاتف المحمول تقوم باستخدامه ؟

- خدمة اس تي سي للدفع (STC Pay)
- خدمة مدى للدفع (Mada Pay)
- خدمة بيان للدفع (Bayan Pay)
- خدمة هللة للدفع Halalah))
- خدمة سامسونق للدفع (Samsung Pay)
- خدمة ابل للدفع (Apple Pay)
- خدمة بي بال للدفع (PayPal)
- خدمة وي تشات للدفع (WeChat pay)
- غير ذلك, الرجــــاء التحديد:________

1. مدة تجربة الاستخدام؟

- أقل من 6 شهور
- بين 6 شهور و 12 شهر
- من سنة إلى سنتين
- فوق سنتين: ____________

1. عدد مرات استخدام محفظة الهاتف المحمول ؟

- مرة في اليوم
- مرة في الأسبوع
- مرة في الشهر
- غير ذلك , الرجــــاء التحديد:_______

**القسم الثاني : آراء المستفيدين من محافظ الهاتف المحمول**

**الرجاء توضيح مستوى موافقتكم على العبارات التالية باختيـار الرقم المناسب أمام كل عبارة :**

| أوافق بشدّة  **SA** | أوافق  **A** | محايد  **N** | لا أوافق  **D** | لا أوافق بشدّة  **SD** |
| --- | --- | --- | --- | --- |
| **5** | **4** | **3** | **2** | **1** |

| مستـــوى الرضا | | | | | العنــاصر | متسلسل |
| --- | --- | --- | --- | --- | --- | --- |
| **SA** | **A** | **N** | **D** | **SD** |  |  |
| 5 | 4 | 3 | 2 | 1 | تتمتع محافظ الهاتف المحمول بمزايا أكثر من أنظمة الدفع عبر الإنترنت أو الدفع النقدي. | RA1 |
| 5 | 4 | 3 | 2 | 1 | تعد محافظ الهاتف المحمول أكثر ملاءمة من أنظمة الدفع عبر الإنترنت أو الدفع النقدي. | RA2 |
| 5 | 4 | 3 | 2 | 1 | تعد محافظ الهاتف المحمول أكثر كفاءة من أنظمة الدفع النقدي أو الإنترنت. | RA3 |
| 5 | 4 | 3 | 2 | 1 | تعد محافظ الهاتف المحمول أكثر فاعلية من أنظمة الدفع عبر الإنترنت أو الدفع النقدي. | RA4 |
| 5 | 4 | 3 | 2 | 1 | استخدام محافظ الهاتف المحمول متوافق مع جميع جوانب أسلوب حياتي. | COMP1 |
| 5 | 4 | 3 | 2 | 1 | استخدام محافظ الهاتف المحمول متوافق تمامًا مع وضعي الحالي. | COMP2 |
| 5 | 4 | 3 | 2 | 1 | أعتقد أن استخدام محافظ الهاتف المحمول يتناسب تمامًا مع الطرق التي أحب الشراء بها. | COMP3 |
| 5 | 4 | 3 | 2 | 1 | استخدام محافظ الهاتف المحمول يناسب أسلوب حياتي. | COMP4 |
| 5 | 4 | 3 | 2 | 1 | من السهل أن تصبح ماهرًا في استخدام محافظ الهاتف المحمول | EOU1 |
| 5 | 4 | 3 | 2 | 1 | التفاعل مع محافظ الهاتف المحمول واضح ومفهوم. | EOU2 |
| 5 | 4 | 3 | 2 | 1 | من السهل اتباع جميع خطوات استخدام محافظ الهاتف المحمول. | EOU3 |
| 5 | 4 | 3 | 2 | 1 | من السهل التفاعل مع محافظ الهاتف المحمول. | EOU4 |
| 5 | 4 | 3 | 2 | 1 | رأيت آخرين يستخدمون محافظ الهاتف المحمول. | OB1 |
| 5 | 4 | 3 | 2 | 1 | رأيت في كثير من الأحيان آخرين يستخدمون محافظ الهاتف المحمول | OB2 |
| 5 | 4 | 3 | 2 | 1 | أعرف الكثير عن المنتجات الجديدة قبل أن يعرفها الآخرون. | TR1 |
| 5 | 4 | 3 | 2 | 1 | أنا بالعادة من أوائل من يجرب المنتجات الجديدة. | TR2 |
| 5 | 4 | 3 | 2 | 1 | أعتقد أن استخدام محافظ الهاتف المحمول سيكون مناسبًا. | CONV1 |
| 5 | 4 | 3 | 2 | 1 | أعتقد أنه من السهل استخدام محافظ الهاتف المحمول لإنجاز عمليات الدفع الخاصة بي. | CONV2 |
| 5 | 4 | 3 | 2 | 1 | استخدام محافظ الهاتف المحمول يحفظ وقتي. | CONV3 |
| 5 | 4 | 3 | 2 | 1 | بالمقارنة مع طرق الدفع التقليدية، أعتقد أن الدفع عن طريق محافظ الهاتف المحمول أكثر ملاءمة. | CONV4 |
| 5 | 4 | 3 | 2 | 1 | إذا سمعت عن تقنية معلومات جديدة ، فساسعى لتجربتها. | PI1 |
| 5 | 4 | 3 | 2 | 1 | من بين أصدقائي / زملائي ، عادةً ما أكون أول من يجرب تقنيات المعلومات الجديدة. | PI2 |
| 5 | 4 | 3 | 2 | 1 | أحب تجربة تقنيات المعلومات الجديدة. | PI3 |
| 5 | 4 | 3 | 2 | 1 | يوفر التطبيق بيئة آمنة لإرسال المعلومات الحساسة من خلاله. | PS1 |
| 5 | 4 | 3 | 2 | 1 | أعتقد أن التطبيق يحتوي على إجراءات أمنية لإرسال المعلومات الحساسة من خلاله. | PS2 |
| 5 | 4 | 3 | 2 | 1 | أعتقد أن تفاصيل العمليات المالية في التطبيق محمية. | PS3 |
| 5 | 4 | 3 | 2 | 1 | سأشعر بالأمان التام عندما استخدم بطاقة الائتمان / الصراف الخاصة بي لشراء المنتجات من خلال التطبيق. | PS4 |
| 5 | 4 | 3 | 2 | 1 | أثق في التطبيق. | PT1 |
| 5 | 4 | 3 | 2 | 1 | أعتقد أن التطبيق دائمًا ما يوفر خدمات مالية دقيقة. | PT2 |
| 5 | 4 | 3 | 2 | 1 | أعتقد أن التطبيق يثير اهتمامي. | PT3 |
| 5 | 4 | 3 | 2 | 1 | أعتقد أن التطبيق يوفر دائمًا خدمات مالية آمنة. | PT4 |
| 5 | 4 | 3 | 2 | 1 | أتوقع أن يزداد استخدامي لمحافظ الهاتف المحمول في المستقبل. | IU1 |
| 5 | 4 | 3 | 2 | 1 | أنوي استخدام محافظ الهاتف المحمول في المستقبل. | IU2 |
| 5 | 4 | 3 | 2 | 1 | إذا أتيحت لي الفرصة ، فسأستخدم محافظ الهاتف المحمول. | IU3 |
| 5 | 4 | 3 | 2 | 1 | سأحاول دائمًا استخدام محافظ الهاتف المحمول. | IU4 |
| 5 | 4 | 3 | 2 | 1 | أخطط لاستخدام محافظ الهاتف المحمول بشكل متكرر. | IU5 |

أشكر لكــم تعـاونكم واقّدر وقتــكم الثمين.
