# Supplementary material for: Determining the factors of m-wallets adoption. A twofold SEM-ANN approach
Source: PLoS One. 2022 Jan 28;17(1):e0262954. doi: 10.1371/journal.pone.0262954 (PMC8797175; doi:10.1371/journal.pone.0262954)
Supplement: Attachment — Submitted filename: Response to Reviewers.docx [file pone.0262954.s005.docx]

**RE: Determining the factors of M-Wallets adoption. A twofold SEM-ANN approach**

Many thanks for undertaking the review on our behalf for the above manuscript. We have meticulously worked through each and every point of the reviewers' concerns and prepared this document to illustrate how we have modified and improved this revision. We hope you will agree that the manuscript has been significantly improved and is now ready for publication in the Journal of Global Information Management.

The manuscript has greatly benefited from the thoughtful insights from the reviewers. As such, we greatly appreciate the editor as well as the reviewers’ time and efforts devoted to improve our work.

Please find the comments, followed by our responses to them.

**Reviewer(s)' Comments to Author:**

# Reviewer: 1

**Comments**

In this paper, the author proposes a study concerning the main characteristics of the adoption of mobile wallets. I have few comments that I think they should be addressed

- line 85: intentions to use m-wallets? -> intentions to use m-wallets?" (missing double quote at the end of research question).

- Improve quality (size, resolution, and dpi) of the figures, especially Figure 1. They are very blurry and can be very hard to understand.

- lines 435-439 you kinda explain what a neural network is, but this section should contain only the findings. You should put a reference to a good paper which explains what is a neural network, rather than including it in a section presenting your experimental results.

**Authors’ Response:**

*Thank you for raising this concern and bringing these points to our attention. In response, we have gone through the paper in details and have incorporated the following revisions accordingly:*

1. *We have added the double quotation marks at the end of research question. Please see page 5, line 106.*
2. *We have revised all the figures to improve the size, resolution, and dpi. The quality of updated figures is better than the earlier figures.*
3. *We have revised Section 4.2 and have removed explanation regarding Artificial Neutral Networks. A single sentence description has been given along with references from good research papers. Please see page 24, lines 464-466.*

# Reviewer: 2

**Comment**
Authors propose a study concerning the adoption of mobile wallets. The paper is interesting, but it is not easy to follow.

First of all, what is the reason behind the choice of the three groups namely technology characteristics? Authors should motivate well this point.

Moreover, I don't think that the description of the dataset could be included in the research methodology. The title of this section leads the knowledge of the idea and how this study is structure, instead it is completely focused on the dataset. I think that this section should be revised.

The proposed model is not well defined and described, and I'm not able to understand the novelty and the differences with the state of the art. Furthermore, why the model is not compared with others in literature?

**Authors’ Response:**

*We are grateful for raising important points. Addressing these concerns will really improve the quality of our paper. In response to the above mentioned comments, we made the following revisions:*

1. *In response to the first comment, the motivation behind the grouping of constructs into three categories have been added to the manuscript. Please see Page 8, Lines 169-174.*
2. *Addressing the second comment, Table-1 (previously), containing the detailed description of the dataset, has been removed. However, a brief description about the sample has been retained in Section 3.2. Please see Pages 16, Lines 333-338.*
3. *To incorporate the third comments, a description about the novelty of our study is given on Pages 4-5 (Lines 84-104). To compare our model with the prior research, a literature review of 15 studies in the context of m-wallets and mobile payment has been presented in Table-1 (newly added). Please see Pages 8-10 (Table-1). Further, Section 2.2 presents detailed description of the model.*

# Response:

*Thank you very much for your constructive feedback. We appreciate you and all the reviewers for raising concerns regarding the various critical issues with the manuscript. In response, we have now worked diligently to address each issue and have provided greater clarity. We hope that you will approve with the additions/revisions made to address the reviewers’ concerns regarding the rigour and theoretical novelty.*

*Best Regards*

*Authors*
